# Associations between normative personality traits and impairments in personality functioning in a clinical adolescent sample

**DOI:** 10.3389/fpsyt.2026.1845402

**Published:** 2026-07-23

**Authors:** Ana Gregorec, Maja Zupančič, Sara Plakolm Erlač, Žiga Kovačič, Hojka Gregorič Kumperščak

**Affiliations:** 1 Unit for Child and Adolescent Psychiatry, Division of Paediatrics, University Medical Centre Maribor, Maribor, Slovenia; 2 Department of Psychology, Faculty of Arts, University of Ljubljana, Ljubljana, Slovenia

**Keywords:** adolescents, dimensional models, ICID, LoPF-Q 12–18, personality functioning, personality traits

## Abstract

Personality disorders are increasingly conceptualized within dimensional models in terms of impairments in personality functioning (identity, self-direction, empathy, and intimacy) and maladaptive personality traits. Adolescence represents a critical developmental period for the emergence of personality pathology; however, empirical studies examining the relationship between personality functioning and normative personality traits in clinical adolescent samples remain scarce. Given that maladaptive traits can be understood as extreme variants of normative personality traits, examining the latter may provide a developmentally sensitive framework for understanding early manifestations of personality pathology. The present study examined impairments in personality functioning and their associations with normative personality trait profiles in a clinical sample of Slovenian adolescents. Participants were 61 adolescents aged 12–19 years receiving inpatient or outpatient psychiatric care. Personality functioning was assessed using the Levels of Personality Functioning Questionnaire for Adolescents (LoPF-Q 12–18), and personality traits were measured using the Inventory of Child Individual Differences (ICID). Descriptive statistics, reliability analyses, correlation analyses, multiple regression analyses, and logistic regression analyses were conducted. Greater impairments in personality functioning were associated with higher neuroticism and both lower conscientiousness and lower extraversion. At the domain level, impairments in identity and self-direction showed the strongest associations with emotional instability and lower self-regulation traits, whereas interpersonal impairments were associated with antagonistic traits and lower social engagement. Logistic regression analyses did not identify significant predictors of broad diagnostic categories. These findings support dimensional approaches to adolescent personality pathology and suggest that impairments in personality functioning are closely linked to emotional vulnerability, reduced self-regulation, and interpersonal difficulties reflected in normative personality traits. Integrating assessments of personality functioning and normative traits may improve clinical understanding of personality pathology in adolescence.

## 1 Introduction

The dimensional perspective on personality disorders (PDs) has gained increasing empirical and theoretical support, as it aligns more closely with the complexity, heterogeneity, and continuity of personality pathology than traditional categorical models (1). Contemporary dimensional frameworks, including the DSM-5 Alternative Model for Personality Disorders (AMPD) and the ICD-11 classification, conceptualize personality pathology in terms of impairments in personality functioning (severity) and maladaptive personality traits (style). Within these models, PDs are understood as maladaptive extremes of shared personality characteristics rather than discrete diagnostic entities (1, 2). Accumulating evidence indicates that impairments in personality functioning—reflecting difficulties in intrapersonal and interpersonal capacities—are the most robust predictors of PD presence, clinical severity, prognosis, and everyday functioning, whereas maladaptive traits primarily describe stylistic expressions of pathology (3–5). Whereas ICD-11 conceptualizes impaired personality functioning as the necessary and sufficient condition for a PD diagnosis, the DSM-5 AMPD requires both impaired functioning and the presence of one or more elevated maladaptive personality traits. The DSM-5 AMPD trait domains—negative affectivity, detachment, antagonism, disinhibition, and psychoticism—show substantial conceptual and empirical overlap with the Five-Factor Model (6), a well-established framework for understanding normative personality variation across the lifespan. Evidence from both adult (7, 8) and adolescent samples (9) indicates that maladaptive personality traits largely represent extreme and negatively valenced variants of normative personality traits rather than qualitatively distinct constructs. This correspondence is particularly evident for negative affectivity, detachment, antagonism, and disinhibition, whereas psychoticism appears to show a less consistent relationship with normative personality dimensions, with evidence suggesting that it does not align closely with openness in the same manner as the other maladaptive trait domains correspond to Five-Factor Model dimensions (9).

Adolescence is widely recognized as a particularly sensitive developmental period for the emergence of personality pathology (10). This period is characterized by rapid neurological and hormonal changes that influence emotion regulation strategies, impulse control, and cognitive control, alongside increasing autonomy and a developmental shift from parents toward peers (11). These changes coincide with heightened instability in thinking, behavior, affect, and self-concept. Within this developmental context, identity formation represents a central task (12, 13), whereby normative identity confusion gradually resolves into a more coherent sense of self as neurodevelopment supports improvements in self-regulation and interpersonal functioning, including empathy and intimacy (14). These developmental processes closely correspond to Criterion A of the DSM-5 AMPD, which captures impairments in identity, self-direction, empathy, and intimacy and provides a key developmental framework for understanding adolescent personality pathology (15).

Sharp’s developmental interpretation of the AMPD proposes that maladaptive personality traits reflect developmental continuity, whereas impairments in personality functioning represent a developmentally discontinuous shift that explains the emergence of PDs during adolescence (15). As adolescents mature, capacities related to identity, self-direction, empathy, and intimacy become increasingly integrated through neurobiological development, advances in metacognitive and mentalizing abilities, and growing social demands. Personality pathology is proposed to arise when this integration fails, resulting in persistent impairments in self and interpersonal functioning. Longitudinal research supports this developmental framework. Impairments in self-functioning increase between ages 12 and 16 and subsequently decline in late adolescence (16), while identity diffusion peaks in early adolescence and decreases into early adulthood (17). Overall, personality pathology tends to emerge in early adolescence, peak in mid-adolescence, and decline into early adulthood for most individuals, although a smaller subgroup shows increasing and persistent difficulties that may reach diagnostic thresholds in adulthood (18–20). Severe impairments in personality functioning, particularly in the domain of the self, tend to remain stable and predict later PD diagnoses (14).

From a developmental trait perspective, broad (or higher-level) personality traits are reliably observable from early childhood onwards, show meaningful rank-order stability over time, and are systematically related to later personality pathology (11, 21, 22). Although early individual differences are often described as temperament, they reflect the same underlying dispositions as later personality traits (23). Empirical evidence supports substantial continuity between childhood temperament and adult personality, alongside gradual differentiation through interactions with the environment (24–26). This developmental continuity provides a strong rationale for examining normative personality traits as early indicators of personality pathology (27). Consistent with this perspective, empirical research demonstrates substantial structural overlap between normative personality traits and maladaptive personality trait domains, indicating that maladaptive traits largely reflect extreme variants of broad personality dimensions (28). For example, negative affectivity corresponds closely to neuroticism, detachment to low extraversion, antagonism to low agreeableness, and disinhibition to low conscientiousness. Lower-order traits may be particularly informative for understanding pathological personality development because they provide more specific information than broad personality dimensions and may help identify developmental pathways to personality pathology (23). Early temperamental and personality characteristics, including emotional reactivity, social withdrawal, and difficulties with self-regulation, have been proposed as developmental risk factors for later personality pathology (23, 27). Normative personality traits also show systematic developmental changes across adolescence, including increases in conscientiousness and openness and decreases in neuroticism (18), whereas maladaptive traits tend to show modest declines, particularly among individuals with lower initial levels (29). Although much of the existing literature has examined normative personality traits as predictors of later personality pathology, the substantial overlap between normative and maladaptive trait dimensions suggests that normative traits may also be informative for understanding current levels of personality functioning.

Several dimensional models have been proposed to describe maladaptive personality traits in youth. De Clercq et al. identified a four-factor structure in children aged 6–14 (emotional instability, disagreeableness, introversion, and compulsivity), organized into higher-order internalizing and externalizing dimensions; in adolescence, this structure expands to a five-factor model with the addition of antagonism (30). Similarly, Tromp and Koot supported a four-dimensional model comprising emotion regulation problems, dissocial behavior, inhibition, and compulsivity (31). Across these models, maladaptive traits are conceptualized as extreme variants of normative personality dimensions. Extending this dimensional perspective within the AMPD framework, See et al. identified hierarchical groups of adolescents with low, moderate, and high levels of maladaptive personality traits, further differentiating subgroups characterized by antagonism, emotional instability, disinhibition, and resilience (32).

Maladaptive traits in childhood have also been shown to contribute to impairments in personality functioning and to represent early vulnerabilities for later psychopathology (33, 34). Interpersonal and emotional difficulties associated with personality traits further illustrate these developmental pathways. For example, shyness is associated with social withdrawal and feelings of loneliness (35), while certain personality characteristics, such as social disengagement, mistrust of others, restricted affectivity (36) or high activity level, high distractibility, low persistence, negative mood (37), may elicit negative responses from peers, such as rejection, thereby hindering the development of reciprocal relationships. Longitudinal research indicates that early emotional instability and self-critical tendencies, both reflecting neuroticism-related characteristics, are strongly associated with later impairments in self-functioning (38). Given that neuroticism is also a trait that typically declines across normative adolescent development (18), elevated levels of neuroticism may reflect deviations from typical developmental trajectories and therefore represent a particularly relevant correlate of impairments in personality functioning.

Consistent with this developmental framework, the DSM-5 AMPD has been proposed as a particularly promising and developmentally sensitive model for assessing personality pathology in adolescence (2). Despite these advances, empirical research simultaneously examining impairments in personality functioning and normative personality traits in clinical adolescent samples remains limited, particularly in smaller European contexts. Integrating dimensional assessments of personality functioning and normative personality traits may provide a developmentally sensitive framework for capturing both the severity and expression of personality pathology. Although maladaptive personality traits are included in contemporary dimensional models of personality pathology, normative personality traits may provide complementary information regarding broader personality processes associated with impairments in personality functioning and may be particularly useful for understanding personality pathology from a developmental perspective.

The present study therefore aimed to examine impairments in personality functioning and their associations with normative personality traits in a clinical sample of Slovenian adolescents. Specifically, the study aimed to describe levels of personality functioning and personality traits and examine associations between impairments in personality functioning and normative personality traits. Based on dimensional models and developmental research, we hypothesized that greater impairments in personality functioning would be associated with less adaptive personality traits, characterized by higher neuroticism and lower conscientiousness, lower agreeableness, and lower extraversion. Given previous findings linking emotional instability to later impairments in self-functioning, we further expected neuroticism to be among the personality dimensions most strongly associated with impairments in personality functioning. Finally, we explored whether impairments in personality functioning and normative personality traits predict differences across broad clinical diagnostic groupings.

## 2 Methods

### 2.1 Participants

The sample included adolescents referred for routine diagnostic assessment and treatment at the University Medical Centre, Division of Pediatrics, Unit for Pediatric and Adolescent Psychiatry. Adolescents with a current psychotic disorder or severe neurodevelopmental disorders, including severe autism spectrum disorder or intellectual disability, were excluded from the study due to the limited applicability of self-report measures. The final sample consisted of 61 adolescents aged 12 to 19 years (M = 15.46, SD = 1.58), of whom 11 (18.0%) were male, 49 (80.3%) were female, and one (1.6%) identified as other.

At the time of data collection, participants were clinically classified into broad diagnostic groupings based on their primary symptom profiles: (1) internalizing disorders (e.g., depressive disorders, anxiety disorders, eating disorders, obsessive–compulsive disorder), (2) externalizing disorders (e.g., conduct disorder, oppositional defiant disorder, attention-deficit/hyperactivity disorder), and (3) mixed/comorbid presentations, defined by the presence of both internalizing and externalizing symptoms and/or emerging personality pathology, including suicidal ideation, non-suicidal self-injury, and marked behavioral dysregulation. Diagnostic classifications were based on clinical evaluations conducted by licensed clinical psychologists and child and adolescent psychiatrists as part of routine care, in accordance with ICD-10 criteria. Given the clinical nature of the sample, diagnostic group sizes were expected to be unequal.

### 2.2 Measures

#### 2.2.1 Personality functioning

Personality functioning was assessed using the *Levels of Personality Functioning Questionnaire for Adolescents* (LoPF-Q 12–18; Goth et al., 2018), a self-report instrument designed to operationalize Criterion A of the DSM-5 Alternative Model for Personality Disorders, which has been validated for use in adolescent samples (39). The questionnaire assesses impairments across four domains of personality functioning: identity, self-direction, empathy, and intimacy, representing core aspects of self and interpersonal functioning. The LoPF-Q 12–18 consists of 97 items rated on a five-point Likert scale (0 = strongly disagree to 4 = strongly agree), with higher scores indicating greater impairment in personality functioning. A total score reflects the overall severity of impairment, whereas domain scores allow for differentiated assessment of self-related (identity, self-direction) and interpersonal (empathy, intimacy) functioning. In addition, the questionnaire provides subdomain scores reflecting more specific aspects of personality functioning within each domain (e.g., self-perception and self-esteem within identity; perspective-taking and prosociality within empathy). The Slovenian version of the LoPF-Q 12–18 has demonstrated good psychometric properties (39). Previous validation studies report excellent internal consistency for the total score (α = .97) and good to excellent reliability for the domain scales (α = .87–.94).

#### 2.2.2 Personality traits

Personality traits were assessed using the *Inventory of Child Individual Differences* (ICID) (40), adapted, validated, and standardized in Slovenia as the VMR-OM (41), and showed sound psychometric properties in different forms (i.e., mother-, father-, teacher, adolescent self-report). The ICID scales assess 15 lower-order personality traits reflecting normative personality characteristics in childhood and adolescence. In Slovenian samples, these scales form four higher-order, relatively modestly related personality dimensions rather than the traditional five-factor structure; instead, the lower-order traits of openness to experience loads on extraversion, and (subjective) intelligence loads on conscientiousness (26, 41). Conscientiousness thus comprises the traits of achievement orientation, compliance, intelligence, organized behavior, and distractibility. Disagreeableness consists of antagonism, negative affect, and strong-will. Neuroticism includes fearfulness–insecurity and shyness. Extraversion comprises activity level, sociability, positive emotions, considerateness, and openness to experience. In the present study, adolescents completed the self-report version of the ICID, which contains 108 items rated on a seven-point Likert-type scale (1 = much less than most peers to 7 = much more than most peers). Higher scores indicate greater expression of the respective personality trait.

### 2.3 Data analysis

Subscale scores for the LoPF-Q 12–18 were calculated as the mean of the respective items after reverse coding when necessary, and a total score was calculated as the mean across all items, with higher scores indicating greater impairment in personality functioning. Missing responses were treated as missing values and excluded from scale calculations. Scores were computed when at least 80% of the items were completed. For the ICID, facet scores were calculated as the mean of the corresponding items. Higher-order personality dimensions were computed following the procedures described in the scoring manual (41) by summing the relevant facet scores; therefore, dimension scores exceed the original 1–7 item scale. For the conscientiousness dimension, the distractibility facet was subtracted from the sum of the remaining facets as specified in the scoring guidelines. Missing responses were again treated as missing values and excluded from scale calculations. Descriptive statistics (means and standard deviations) were calculated for LoPF-Q 12–18 total scores, domains, and subdomains, as well as for ICID personality traits and higher-order dimensions. Internal consistency of the scales was evaluated using Cronbach’s alpha coefficients. Pearson correlation analyses were conducted to examine associations between impairments in personality functioning and normative personality traits. Correlations were calculated between LoPF-Q 12–18 (total and domain scores) and ICID (higher- and lower-order trait scores). To examine the unique contributions of personality dimensions to impairments in personality functioning, a multiple linear regression analysis was conducted with LoPF-Q total score as the dependent variable and the four ICID higher-order personality dimensions as predictors.

Due to the unequal distribution of participants across diagnostic categories and the small number of cases in several groups, the diagnostic variable was recoded for regression analysis to ensure sufficient group sizes and more stable statistical estimation. Specifically, adolescents with comorbid presentations, including those with emerging personality pathology, were grouped together and contrasted with adolescents with internalizing disorders. Given the very small number of cases with predominantly externalizing presentations (n = 2), these participants were added to the personality pathology/comorbid group to avoid the formation of an additional underpowered category. This grouping approach was determined prior to conducting the regression analyses and was primarily driven by sample size considerations to ensure sufficient statistical power and model stability. It should therefore be interpreted as a pragmatic analytic decision rather than a theoretically defined classification. This recoding resulted in two diagnostic groups suitable for logistic regression analysis: an internalizing group and a personality pathology/comorbid group.

A binary logistic regression analysis was conducted to examine whether impairments in personality functioning and personality traits predicted diagnostic group membership. Predictor variables included the LoPF-Q 12–18 total score and the four ICID higher-order personality dimensions (neuroticism, extraversion, disagreeableness, and conscientiousness). To complement the regression analysis and examine diagnostic group differences without simultaneously controlling for shared variance among predictors, independent-samples t-tests were conducted comparing the internalizing and personality pathology/comorbid group on LoPF-Q 12–18 total score and the four ICID higher-order personality dimensions. Assumptions of normality and homogeneity of variance were examined prior to analysis. All statistical analyses were conducted using IBM SPSS Statistics (Version 26). Statistical significance was evaluated using two-tailed tests with *α* = .05.

## 3 Results

### 3.1 Descriptive statistics and internal reliability

Descriptive statistics and internal consistency estimates for the LoPF-Q 12–18 scales are presented in **Table 1**. Internal consistency of the LoPF-Q 12–18 scales was good to excellent, with excellent reliability for the total score and good to excellent reliability across domains and subdomains.

**Table 1:** Descriptive statistics for LoPF-Q 12-18.

LoPF-Q 12–18 Scale	Mean	SD	Cronbach’s α
Total Score	1.96	0.59	.961
Identity	2.17	0.70	.872
Identity continuity	2.11	0.75	.793
Identity coherence	2.22	0.76	.780
Self-direction	2.45	0.83	.936
Self-direction congruence	2.76	0.91	.895
Self-direction purpose	2.21	0.84	.888
Empathy	1.28	0.58	.872
Empathy perspective	1.60	0.76	.782
Empathy prosocial	1.08	0.60	.820
Intimacy	1.94	0.66	.886
Intimacy closeness	2.37	0.70	.753
Intimacy reciprocity	1.68	0.74	.857

N = 61. LoPF-Q 12-18 = Levels of Personality Functioning Questionnaire. Higher scores indicate greater expression of the respective personality functioning scale.

Descriptive statistics and internal consistency estimates for the ICID lower-order personality traits and dimensions are presented in **Table 2**. Internal consistency of the higher-order personality dimensions ranged from acceptable to good. Reliability estimates for lower-order traits were more variable, ranging from low to excellent. The highest internal consistency was observed for sociability, positive emotions, activity level, and considerateness, whereas achievement orientation and distractibility showed the lowest reliability coefficients. Lower reliability of some lower-order scales compared to the representative Slovenian sample (41) may have attenuated associations with personality functioning and should therefore be interpreted with caution, particularly at the lower-order trait level.

**Table 2:** Descriptive statistics for ICID.

ICID Trait/Dimension	Mean	SD	Cronbach’s α
Dimensions			
Neuroticism	8.74	2.00	.602
Extraversion	21.42	3.80	.743
Disagreeableness	10.59	2.20	.583
Conscientiousness	13.43	3.20	.773
Lower-order traits			
Achievement orientation	4.52	0.81	.242
Compliant	4.59	0.84	.619
Intellect	4.11	0.75	.562
Organized	4.48	1.10	.788
Distractible	4.27	0.89	.466
Antagonism	2.50	0.78	.666
Negative affect	4.43	1.25	.784
Strong–willed	3.66	0.88	.583
Fearful–insecure	4.45	1.16	.729
Shy	4.30	1.21	.718
Activity level	3.81	1.30	.798
Sociable	3.73	1.18	.870
Openness	4.32	0.79	.592
Positive emotions	4.35	1.12	.828
Considerate	5.21	0.95	.798

N = 61. ICID = Inventory of Child Individual Differences. Higher scores indicate greater expression of the respective personality trait.

### 3.2 Associations between personality functioning and personality traits

To examine associations between impairments in personality functioning and normative personality traits, Pearson correlation analyses were conducted between LoPF-Q 12–18 scores and ICID scores. The full correlation matrix between LoPF-Q 12–18 domains and ICID personality traits (lower-order traits and dimensions) is presented in **Supplementary Table S1**. Overall, impairments in personality functioning were associated with a pattern of personality traits consistent with theoretical expectations from dimensional models of personality pathology. Higher levels of impairment (LoPF-Q 12–18 Total Score) were significantly associated with higher neuroticism (*r* = .49, p <.001) and both lower conscientiousness (*r* = −.56, *p* <.001) and lower extraversion (*r* = −.38, *p* = .002). At the trait level, impairments in personality functioning were positively associated with maladaptive emotional traits such as fearful–insecure (*r* = .49, *p* <.001), negative affect (*r* = .41, *p* <.01), and distractibility (*r* = .54, *p* <.001), and negatively associated with adaptive traits reflecting self-regulation and positive engagement with the environment, including organized (*r* = −.44, *p* <.001), subjective intelligence (*r* = −.44, *p* <.001), activity level (*r* = −.36, *p* = .004), sociability (*r* = −.36, *p* = .004), and positive emotions (*r* = −.48, *p* <.001). Similar patterns were observed across the LoPF-Q 12–18 domains, particularly for identity and self-direction, which showed consistent associations with neuroticism-related traits and with lower levels of conscientiousness-related traits. Interpersonal impairments also demonstrated theoretically coherent patterns: higher levels of empathy impairment were positively associated with antagonism (*r* = .48, *p* <.001) and disagreeableness (*r* = .45, *p* <.001), whereas higher levels of intimacy impairment showed associations with neuroticism-related traits and lower levels of extraversion-related traits. Taken together, the pattern of correlations indicates that greater impairments in personality functioning are characterized by elevated emotional instability and reduced adaptive personality traits related to self-regulation, organization, and positive social engagement. Overall, the pattern of correlations supported the study hypotheses, indicating that impairments in personality functioning were associated with higher neuroticism and lower levels of both conscientiousness and extraversion. To examine whether the observed associations were independent of shared variance among personality dimensions, a multiple linear regression analysis was conducted with impairments in personality functioning (LoPF total score) as the dependent variable and the four ICID personality dimensions as predictors (**Supplementary Table S2**). The overall model was statistically significant, F(4,56) = 9.90, p <.001, explaining 41.4% of the variance in personality functioning (R² = .41, adjusted R² = .37). Higher neuroticism (β = .34, p = .011) and lower conscientiousness (β = −.46, p = .002) independently predicted greater impairments in personality functioning, whereas extraversion and disagreeableness did not contribute significantly to the model.

### 3.3 Diagnostic group comparisons

To examine whether the recoded diagnostic groups differed on personality functioning and personality trait dimensions without controlling for shared variance among predictors, independent-samples t-tests were conducted. No statistically significant differences were observed between the internalizing and personality pathology/comorbid group on LoPF total score, neuroticism, extraversion, disagreeableness, or conscientiousness (all p’s >.05). The largest difference was observed for personality functioning, with adolescents in the personality pathology/comorbid group reporting higher levels of impairment (M = 2.09, SD = 0.51) than those in the internalizing group (M = 1.82, SD = 0.64), although this difference did not reach statistical significance, t(59) = -1.82, p = .073. The corresponding effect size was in the small-to-medium range (Cohen’s d = 0.47).

### 3.4 Prediction of diagnostic group

A binary logistic regression analysis was conducted to examine whether impairments in personality functioning and normative personality traits predicted membership in the recoded diagnostic groups (internalizing vs. PD/comorbid). The overall model was not statistically significant, *χ²*(5) = 5.88, *p* = .318, and explained a modest proportion of variance (Nagelkerke *R²* = .13). None of the individual predictors reached statistical significance. Odds ratios for all predictors were close to 1, and their confidence intervals included 1, indicating that none of the predictors showed reliable effects on diagnostic group membership. Although higher levels of personality functioning impairment and neuroticism were associated with increased odds of belonging to the personality pathology/comorbid group, and higher conscientiousness was associated with lower odds, these effects did not reach statistical significance. These findings suggest that, within the present sample, personality functioning and personality traits were not strong independent predictors of diagnostic group.

## 4 Discussion

The present study examined impairments in personality functioning and their associations with normative personality traits in a clinical sample of Slovenian adolescents. Consistent with contemporary dimensional models of personality pathology, the findings demonstrated systematic associations between impairments in personality functioning and normative personality traits. Specifically, greater impairments in personality functioning were associated with higher levels of neuroticism and lower levels of conscientiousness and extraversion. These findings extend existing research by demonstrating that, even within a clinically referred adolescent sample, impairments in personality functioning are embedded within normative personality trait structures and can be meaningfully linked to specific domains of self and interpersonal functioning. This supports the view that personality pathology in adolescence reflects maladaptive configurations of general personality dimensions rather than qualitatively distinct categories (2, 8). Moreover, the results highlight the clinical relevance of integrating assessments of personality functioning and normative personality traits, as these dimensions appear to capture complementary aspects of severity and stylistic expression within adolescent personality pathology. It should be noted that some lower-order ICID traits demonstrated relatively low internal consistency, which may have reduced the strength of trait-level associations; however, higher-order personality dimensions showed more acceptable reliability, supporting the robustness of the main findings.

One of the most consistent findings was the association between impairments in personality functioning and traits reflecting emotional instability. Higher levels of neuroticism, fearful–insecure tendencies, and negative affect were associated with greater impairment, particularly within the domains of identity and self-direction. This pattern suggests that emotional instability may play a central role in disrupting the development of coherent self-functioning during adolescence, rather than merely co-occurring with personality pathology. From a developmental perspective, heightened emotional reactivity may amplify self-doubt, increase sensitivity to stress, and interfere with the integration of stable goals and values. This interpretation aligns with models of personality pathology emphasizing the role of emotional vulnerability and emotion regulation difficulties in impairments in self-functioning (15, 33). It is also consistent with previous research demonstrating strong associations between neuroticism-related traits and personality pathology across adolescence and adulthood (8, 9).

In addition to emotional instability, impairments in personality functioning were strongly associated with lower levels of traits reflecting self-regulation and goal-directed behavior. Conscientiousness and several of its underlying facets, including organized behavior and subjective intelligence, showed robust negative associations with impairments, particularly within the domain of self-direction. These findings are conceptually consistent with Criterion A, as self-direction involves the capacity to pursue meaningful goals, regulate behavior, and maintain internal standards (15). From a developmental perspective, lower conscientiousness may reflect difficulties in planning, sustaining effort, and organizing behavior, which can interfere with the formation and maintenance of coherent goals. Such difficulties may reduce adolescents’ ability to translate intentions into consistent action, thereby contributing to impairments in personality functioning. This interpretation aligns with research linking deficits in self-regulatory capacities to personality pathology (11, 22, 27). This interpretation is further supported by the multiple regression analysis, which indicated that neuroticism and conscientiousness remained independently associated with impairments in personality functioning after accounting for the other personality dimensions. In contrast, extraversion and disagreeableness did not explain unique variance in personality functioning beyond their shared associations with the other personality traits. These findings suggest that emotional instability and self-regulatory capacities may represent particularly important and independent dimensions underlying impairments in personality functioning during adolescence.

The interpersonal domains of personality functioning also demonstrated theoretically meaningful associations with personality traits. Impairments in empathy were most strongly associated with antagonism and disagreeableness, suggesting that adolescents with elevated antagonistic traits may have difficulty recognizing and responding to the emotional experiences of others. This pattern is consistent with models linking antagonism to deficits in perspective-taking and reduced prosocial orientation (8, 34, 42). In contrast, impairments in intimacy were more closely associated with traits reflecting lower social engagement and positive affect, including lower extraversion and its lower-order traits of sociability and positive emotions. This is consistent with previous findings linking lower social engagement and shyness to difficulties in forming close relationships and increased feelings of loneliness (35, 37). This distinction suggests that different interpersonal impairments may arise from partially distinct trait-based pathways: antagonistic tendencies may disrupt empathy through reduced concern for others, whereas low extraversion may hinder the formation of close relationships through reduced social approach and engagement. Clinically, this may manifest as qualitatively different interpersonal difficulties, with some adolescents presenting as interpersonally insensitive or conflict-prone, and others as socially withdrawn or disengaged. These findings highlight the importance of differentiating between interpersonal domains when assessing personality functioning in adolescence.

Taken together, the correlation and regression findings suggest two broad developmental mechanisms underlying impairments in personality functioning. First, impairments appear to be associated with elevated emotional vulnerability, reflected in higher levels of neuroticism-related traits (15, 38). Second, impairments are linked to reduced capacities for self-regulation and goal-directed behavior, reflected in lower levels of conscientiousness-related traits (11, 22). Although these mechanisms are conceptually distinct, they may also interact, as heightened emotional reactivity can further undermine self-regulatory capacities during adolescence. Together, these findings support the view that personality pathology reflects maladaptive extremes of normative personality characteristics rather than a categorically distinct phenomenon (2, 8).

In contrast to the relatively strong associations observed in the correlation analyses, the logistic regression analysis did not identify significant independent predictors of diagnostic group membership when impairments in personality functioning and personality traits were considered simultaneously. Although the direction of the effects was consistent with theoretical expectations—higher levels of impairment and neuroticism were associated with greater likelihood of belonging to the personality pathology/comorbid group, whereas higher conscientiousness was associated with lower likelihood—these effects did not reach statistical significance. One interpretation is that categorical diagnostic groupings may capture heterogeneous clinical presentations that do not map closely onto underlying dimensional personality processes (4, 5). In this sense, the lack of significant predictors may reflect a mismatch between categorical classifications and the continuous nature of personality functioning and trait variation in adolescence. At the same time, the relatively small sample size may have limited statistical power to detect independent effects within the regression model. Consistent with this interpretation, exploratory group comparisons between the internalizing and personality pathology/comorbid group also did not reveal statistically significant differences in personality functioning or personality trait dimensions. Although adolescents in the personality pathology/comorbid group reported somewhat higher levels of personality functioning impairment, the effect did not reach statistical significance. Together, these findings suggest substantial overlap in personality functioning and personality trait profiles across the recoded diagnostic groups. These findings are broadly consistent with dimensional approaches to personality pathology, although this interpretation should be considered cautiously given the sample size and *post hoc* diagnostic grouping in the present study.

Overall, the present findings are consistent with dimensional models of personality pathology and underscore the value of integrating assessments of personality functioning and normative personality traits in adolescence (2, 5). Importantly, the results indicate that impairments in personality functioning are closely linked to broader patterns of emotional vulnerability, self-regulation, and interpersonal functioning. This supports the view that personality pathology in adolescence reflects maladaptive configurations of normative personality dimensions rather than qualitatively distinct categories (15, 22). By linking impairments in personality functioning with normative trait profiles in a clinical sample, the present study contributes to a more developmentally grounded understanding of personality pathology and highlights the potential utility of combining these approaches in clinical assessment.

## 5 Clinical implications

The present findings have several clinical implications. First, the observed associations between impairments in personality functioning and normative personality traits suggest that incorporating trait-based assessments alongside measures of personality functioning may enhance clinical evaluation in adolescents. In particular, elevated emotional instability and reduced self-regulatory capacities appear to be closely linked to impairments in identity and self-direction (15, 16, 38). Clinically, this may manifest as unstable self-concept, inconsistent goals, and difficulties with planning and follow-through. Identifying such patterns may help clinicians recognize adolescents who are at increased risk for more severe or persistent personality pathology and may benefit from early, targeted intervention.

Second, the findings highlight the value of dimensional assessment approaches. Although diagnostic group membership was not significantly predicted in the regression analyses, the observed associations between impairments in functioning and personality trait profiles suggest that categorical diagnoses may not fully capture clinically relevant variation (4, 5). Integrating dimensional assessments of personality functioning and traits may therefore support more nuanced case conceptualization, particularly in complex or comorbid presentations. This may be particularly useful in early stages of assessment, where trait patterns can provide initial indicators of underlying personality dysfunction before full diagnostic clarity is established.

Finally, the findings underscore the importance of early identification and intervention during adolescence, a developmental period in which personality functioning is still consolidating (14, 18). Difficulties in emotional regulation, self-regulation, and interpersonal functioning may signal emerging vulnerabilities that can be targeted through intervention. Approaches focusing on strengthening emotion regulation, identity development, and interpersonal skills may be particularly relevant. Overall, integrating information on personality functioning and normative personality traits may support more individualized assessment and intervention planning in clinical practice.

## 6 Limitations

Several limitations of the present study should be acknowledged. First, the sample size was relatively small, particularly when examining differences between diagnostic groups. This may have limited statistical power to detect independent predictors in the logistic regression analyses and contributed to the modest explanatory power of those models. Although the multiple regression analysis identified significant independent associations between personality dimensions and personality functioning, the relatively small sample may have reduced the precision of parameter estimates and limited the detection of smaller effects. Accordingly, findings related to diagnostic group comparisons should be interpreted with caution.

Second, the diagnostic groups were heterogeneous and unequally distributed. Some categories included only a small number of participants, necessitating the collapsing of diagnostic groups for regression analyses. Although this approach improved statistical stability, it may have reduced the specificity with which diagnostic differences could be examined and limited interpretability, as the resulting groups do not represent theoretically coherent diagnostic constructs. An additional limitation is the absence of a non-clinical comparison group, which restricts the ability to determine whether the observed patterns are specific to clinically referred adolescents or reflect broader developmental variation.

Third, the study relied exclusively on self-report measures of personality functioning and personality traits. Such assessments may be influenced by response biases or limited self-insight, particularly in adolescent samples (34). Future research would benefit from incorporating multi-informant approaches, including parent reports, clinician ratings, and behavioral or observational measures, to provide a more comprehensive assessment of personality functioning.

Finally, the cross-sectional design precludes conclusions about developmental processes or causal relationships between personality traits and impairments in personality functioning. Longitudinal studies are needed to clarify how these constructs interact over time and to examine the developmental pathways underlying personality pathology (14, 18). In addition, replication in larger and more diverse clinical samples would be important to establish the generalizability of the present findings.

## 7 Future research

Future research should aim to extend the present findings by examining the developmental interplay between personality functioning and normative personality traits using longitudinal designs. Future studies with larger samples would be needed to examine whether similar patterns emerge across more differentiated diagnostic groupings.

Longitudinal studies (e.g., cross-lagged panel designs) may help clarify how impairments in personality functioning and personality trait profiles evolve across adolescence and early adulthood. Such designs could clarify whether specific personality traits represent early indicators of risk for the development of personality pathology or whether impairments in personality functioning contribute to subsequent changes in personality traits over time (14, 18). In addition, longitudinal approaches would allow for a more direct examination of the developmental mechanisms suggested in the present study, particularly the interplay between emotional vulnerability and self-regulatory capacities.

Future research would also benefit from including non-clinical comparison groups to better distinguish between normative developmental variation and clinically significant impairment. This would help clarify the extent to which the observed associations between personality functioning and trait expressions are specific to clinically referred adolescents.

Finally, integrating dimensional models of personality pathology with broader developmental frameworks may provide a more comprehensive understanding of personality development in adolescence. Future studies should consider the role of environmental and contextual factors, including peer relationships, family dynamics, and broader social influences (11, 34). Examining how these contextual factors interact with personality functioning and personality traits may help to clarify the developmental pathways leading to personality pathology and inform more targeted prevention and intervention strategies. Person-centered approaches, such as latent profile or cluster analyses, may also be useful for identifying distinct configurations of personality functioning and trait profiles in adolescent samples.

## Data Availability

The raw data supporting the conclusions of this article will be made available by the authors, without undue reservation.

## Supplementary material

Supplementary Table 1 Pearson correlations between LoPF-Q 12–18 domains and ICID personality traits (dimensions and lower-order traits). Variable labels. 1 = LoPF-Q 12–18 Total Score; 2 = Identity; 3 = Self-direction; 4 = Empathy; 5 = Intimacy; 6 = Achievement orientation; 7 = Compliant; 8 = Intellect; 9 = Organized; 10 = Distractible; 11 = Antagonism; 12 = Negative affect; 13 = Strong-willed; 14 = Fearful–insecure; 15 = Shy; 16 = Activity level; 17 = Sociable; 18 = Openness; 19 = Positive emotions; 20 = Considerate; 21 = Neuroticism; 22 = Extraversion; 23 = Disagreeableness; 24 = Conscientiousness. N = 61. LoPF-Q 12–18 = Levels of Personality Functioning Questionnaire. ICID = Inventory of Child Individual Differences. Values are Pearson correlation coefficients. Only the lower triangle of the correlation matrix is displayed. p < .05, p < .01, p < .001.

Supplementary Table 2 Multiple linear regression predicting impairments in personality functioning (LoPF-Q 12–18 total score) from ICID personality dimensions. N = 61. LoPF-Q 12–18 = Levels of Personality Functioning Questionnaire; ICID = Inventory of Child Individual Differences; B = unstandardized regression coefficient; SE B = standard error of B; β = standardized regression coefficient; CI = confidence interval. Model statistics: R = .644, R^2^ = .414, adjusted R^2^ = .372, F(4,56) = 9.90, p < .001.
