# Supplementary material for: Associations between normative personality traits and impairments in personality functioning in a clinical adolescent sample
Source: Front Psychiatry. 2026 Jul 23;17:1845402. doi: 10.3389/fpsyt.2026.1845402 (PMC13443301; doi:10.3389/fpsyt.2026.1845402)
Supplement: Supplementary file 1 [file Table1.docx]

| **Supplementary Table S1. Pearson correlations between LoPF-Q 12–18 domains and ICID personality traits (dimensions and lower-order traits)** | | | | | | |
| --- | --- | --- | --- | --- | --- | --- |
| **Variable** | | **1** | **2** | **3** | **4** | **5** |
| 1 | LoPF-Q 12-18 Total Score | — |  |  |  |  |
| 2 | Identity | .91*** | — |  |  |  |
| 3 | Self-direction | .89*** | .81*** | — |  |  |
| 4 | Empathy | .74*** | .56*** | .48*** | — |  |
| 5 | Intimacy | .85*** | .70*** | .66*** | .56*** | — |
| 6 | Achievement orientation | −.25* | −.21 | −.27* | −.16 | −.20 |
| 7 | Compliant | −.35** | −.24 | −.30* | −.45*** | −.23 |
| 8 | Intellect | −.44*** | −.36** | −.45*** | −.17 | −.49*** |
| 9 | Organized | −.44*** | −.32* | −.40** | −.41** | −.37** |
| 10 | Distractible | .54*** | .46*** | .55*** | .37** | .45*** |
| 11 | Antagonism | .18 | .06 | .04 | .48*** | .11 |
| 12 | Negative affect | .41** | .49*** | .40** | .36** | .16 |
| 13 | Strong–willed | −.13 | −.11 | −.24 | .19 | −.22 |
| 14 | Fearful–insecure | .49*** | .52*** | .55*** | .21 | .35** |
| 15 | Shy | .33* | .26* | .34** | .10 | .40** |
| 16 | Activity level | −.36** | −.41** | −.43** | −.05 | −.29* |
| 17 | Sociable | −.36** | −.31* | −.43*** | −.03 | −.41*** |
| 18 | Openness | .04 | .04 | −.05 | .24 | −.05 |
| 19 | Positive emotions | −.48*** | −.44*** | −.48*** | −.35** | −.33** |
| 20 | Considerate | −.06 | .07 | .03 | −.29* | −.08 |
| 21 | Neuroticism | .49*** | .46*** | .53*** | .19 | .45*** |
| 22 | Extraversion | −.38** | −.33** | −.42*** | −.15 | −.35** |
| 23 | Disagreeableness | .24 | .25 | .14 | .45*** | .04 |
| 24 | Conscientiousness | −.56*** | −.44*** | −.54*** | −.44*** | −.48*** |
| **Variable labels.** 1 = LoPF-Q 12–18 Total Score; 2 = Identity; 3 = Self-direction; 4 = Empathy; 5 = Intimacy; 6 = Achievement orientation; 7 = Compliant; 8 = Intellect; 9 = Organized; 10 = Distractible; 11 = Antagonism; 12 = Negative affect; 13 = Strong-willed; 14 = Fearful–insecure; 15 = Shy; 16 = Activity level; 17 = Sociable; 18 = Openness; 19 = Positive emotions; 20 = Considerate; 21 = Neuroticism; 22 = Extraversion; 23 = Disagreeableness; 24 = Conscientiousness.  **Note.** N = 61. LoPF-Q 12–18 = Levels of Personality Functioning Questionnaire. ICID = Inventory of Child Individual Differences. Values are Pearson correlation coefficients. Only the lower triangle of the correlation matrix is displayed. *p* < .05, **p** < .01, ***p*** < .001. | | | | | | |
