# Supplementary material for: Associations between normative personality traits and impairments in personality functioning in a clinical adolescent sample
Source: Front Psychiatry. 2026 Jul 23;17:1845402. doi: 10.3389/fpsyt.2026.1845402 (PMC13443301; doi:10.3389/fpsyt.2026.1845402)
Supplement: Supplementary file 2 [file Table2.docx]

| **Supplementary Table S2. Multiple linear regression predicting impairments in personality functioning (LoPF-Q 12–18 total score) from ICID personality dimensions** | | | | | | |
| --- | --- | --- | --- | --- | --- | --- |
| **Predictor** | **B** | **SE B** | **β** | **t** | **p** | **95% CI for B** |
| Constant | 1.783 | 0.633 | — | 2.819 | .007 | [0.516, 3.051] |
| Neuroticism | 0.107 | 0.041 | 0.337 | 2.634 | .011 | [0.026, 0.188] |
| Extraversion | 0.009 | 0.025 | 0.056 | 0.355 | .724 | [−0.041, 0.058] |
| Disagreeableness | 0.015 | 0.033 | 0.055 | 0.451 | .654 | [−0.052, 0.082] |
| Conscientiousness | −0.085 | 0.026 | −0.461 | −3.280 | .002 | [−0.137, −0.033] |
| **Note.** N = 61. LoPF-Q 12–18 = Levels of Personality Functioning Questionnaire; ICID = Inventory of Child Individual Differences; B = unstandardized regression coefficient; SE B = standard error of B; β = standardized regression coefficient; CI = confidence interval. **Model statistics**: R = .644, R² = .414, adjusted R² = .372, F(4,56) = 9.90, p < .001. | | | | | | |
